# From the Clinic to the Laboratory, and Back Again: Investigations on Cannabinoids and Endocannabinoid System Modulators for Treating Schizophrenia

**DOI:** 10.3389/fpsyt.2021.682611

**Published:** 2021-07-05

**Authors:** Kurt Leroy Hoffman

**Affiliations:** Centro de Investigación en Reproduccion Animal, Universidad Autónoma de Tlaxcala-Centro de Investigación y de Estudios Avanzados del Instituto Politécnico Nacional (CINVESTAV'-IPN), Tlaxcala, Mexico

**Keywords:** schizophrenia, animal model, cannabidiol, endocannabinoid, anandamide

## Abstract

The present mini-review focuses on animal models of schizophrenia that have explored the effects of cannabidiol (CBD; a non-psychoactive component of cannabis) or the pharmacological manipulation of the endocannabinoid system on behavioral and cognitive outcome measures. First, results of some relevant clinical studies in this area are summarized, and then pre-clinical work on animal models of schizophrenia based on NMDA receptor antagonism or neurodevelopmental manipulations are discussed. A brief overview is given of the theoretical framework on which these models are based, along with a concise summary of results that have been obtained. Clinical results using CBD for schizophrenia seem promising and its effects in animal models of schizophrenia support its potential as a useful pharmacotherapy. Animal models have been paramount for elucidating the actions of CBD and the function of the endocannabinoid system and for identifying novel pharmacological targets, such as cannabinoid receptors and anandamide. However, more attention needs to be placed on defining and applying independent variables and outcome measures that are comparable between pre-clinical and clinical studies. The objective of this review is, on the one hand, to emphasize the potential of such models to predict clinical response to experimental drugs, and on the other hand, to highlight areas in which research on such models could be improved.

## Introduction: From Anecdote to Clinical Trials and Animal Models

During the 1940s−50s, the principle bioactive components of cannabis–cannabinoids–were identified as delta-9 tetrahydrocannabinol (THC; the psychoactive component) and cannabidiol (CBD), through the work of Adams et al. in the USA, and Todd et al. in Great Britain [reviewed in (1)]. In the 1980s−90s, the discovery of endogenous cannabinoid receptors in the nervous system (2, 3) and their endogenous ligands anandamide and 2-arachidonylglycerol (2-AG) (4, 5) provided a physiological framework within which to investigate the psychoactive properties of THC, the possible therapeutic value of cannabinoids, and the function of the endocannabinoid system. In the context of schizophrenia pharmacotherapy, most clinical investigations have focused on CBD.

The first published report of CBD as a possible treatment for schizophrenia was a case study of a 19-year old female patient, whose symptoms were reduced by a 4-week CBD treatment [1,200 mg/day; (6)]; however, results of later case studies were equivocal (7). More recently, studies showing positive results include a double-blind randomized clinical trial comparing CBD (20 patients) to amisulpride [19 patients; (8)]. Dosing of CBD began at 200 mg/day, was raised to 800 mg/day during the first week and was maintained at that level for 3 weeks. CBD was equally as effective as amisulpride, both showing significant reductions in PANSS positive, negative, and total scores. In CBD treated patients, changes in PANSS total scores were negatively associated with increases in serum anandamide, suggesting that therapeutic effects of CBD were related to an inhibition of anandamide degredation. More recent studies indicate that CBD blocks human fatty acid amide hydrolase binding proteins (FABPs), thereby preventing transport of anandamide to fatty acid amide hydrolase (FAAH, the enzyme that degrades anandamide) (9). A second study was a randomized, double-blind, placebo controlled study comparing CBD (1,000 mg/day; 42 patients) to placebo (40 patients) as an add-on to their ongoing antipsychotic treatment (10). CBD reduced PANSS positive, but not negative or total, symptom scores. Executive function, assessed by the Brief Assessment of Cognition in Schizophrenia (BACS), showed a nearly significant improvement in the CBD group (*p* = 0.068). By contrast, a double-blind, placebo-controlled study of CBD (600 mg/day) as an add-on to ongoing antipsychotic found no effect of CBD on PANSS scores or on cognitive symptoms (11). Apart from CBD dosing, these two studies differed in ethnic composition, being predominantly European Caucasian in the McGuire study vs. a more mixed race population in the Boggs study. Additionally, there could have been important differences in CBD-antipsychotic interactions, if the two patient populations differed with respect to specific antipsychotics used. Finally, responsiveness of positive symptoms to CBD in the McGuire study was quite modest, and while CBD reduced positive symptoms similarly in the Boggs study, there was a significant placebo effect. Three randomized, double-blind, placebo controlled studies tested rimonabant [a cannabinoid receptor type 1 (CB1) inverse agonist]. Two of these studies, one testing rimonabant alone [20 mg/day; (12)] and the other as an add-on to ongoing antipsychotic treatment in overweight patients [20 mg/day; (13)] found no significant effects of rimonabant on positive or negative (12), or cognitive (13) symptoms. A third randomized pilot study (14) in a small group of overweight patients reported that rimonabant (20 mg/day) had no effect on negative symptoms, but improved anxiety and hostility subscales of the Brief Psychiatric Rating Scale (14).

## Experimental Paradigms for Modeling Schizophrenia in Laboratory Rodents: Independent and Dependent Variables

Although the “classical” criteria of face, predictive, and construct validity are those most often applied for evaluating animal models of neuropsychiatric disorders (15), another useful context for evaluating an animal model is to consider it in terms of an experimental paradigm having a set of independent and dependent variables (16–18). Independent variables include species and sex of the subjects, their genetic characteristics, and the experimental manipulation applied. Dependent variables include quantifiable neurobiological or behavioral endpoints. An assessment of the model would first critically consider whether the model's independent and dependent variables are homologous to known pathogenic risk factors and psychiatric symptoms, respectively. A second consideration would be whether the relationship between the independent and dependent variables corresponds to the real-life relationship between risk factor and psychiatric symptoms (“inductive validity”) (17). Regardless of the specific criteria of validity that are applied, such criteria should be considered as a means to define the strengths and limitations of the model in order to provide a context within which to critically interpret the results that the model generates.

In addition to (and often distinct from) modeling the pathophysiology of a psychiatric disorder, an important *practical* function of an animal model is to accurately predict pharmacological responsiveness in the clinic, or “predictive validity” (15). This mini-review will focus on pre-clinical studies that have investigated CBD or pharmacological manipulations of the endocannabinoid system in animal models of schizophrenia, with the following objectives: (1) determine how well animal models of schizophrenia predicted or corresponded to clinical responsiveness, and (2) evaluate these same animal models within the context of inductive validity, as defined above.

### Behavioral Outcome Measures (Dependent Variable)

Behavioral outcome measures that have been considered in studies of CBD or pharmacological manipulation of the endocannabinoid as possible treatments for schizophrenia include pre-pulse inhibition (PPI), tests of cognition, and tests of social deficits.

#### Pre-pulse Inhibition

Operationally, PPI is the capacity of a non-startling auditory pre-stimulus to inhibit the startle response to a startling auditory stimulus delivered 30–500 ms later. People with schizophrenia, as well as their first-degree relatives, show a reduction in this measure (19). Reduced PPI is an indication of deficient sensorimotor gating, and can be easily measured in both humans and in non-human animals.

#### Cognition

The MATRICS initiative (Measurement and Treatment to Improve Cognition in Schizophrenia) launched by the National Institute of Mental Health (NIMH) defined seven cognitive domains that are disrupted in schizophenia: working memory, visual learning and memory, verbal learning and memory, processing speed, attention and vigilance, reasoning and problem solving, and social cognition (20, 21). A complementary initiative, the Cognitive Neuroscience Treatment Research to Improve Cognition in Schizophrenia (CNTRICS) identified translational cognitive tasks with construct validity that can be applied in humans and in rodent models (22). These tasks encompass cognitive domains of attention, cognitive control, declarative memory, reward learning, action selection/preference based decision making (23).

In practice, one of the most frequently applied cognitive tests in animal models of schizophrenia is the novel object recognition (NOR) test, which is considered a test of episodic memory (24). In general, this test comprises a habituation phase, in which the rodent is allowed to explore two objects. After a brief inter-trial interval, the animal is exposed to one of the original objects along with a novel object. The unconditioned response of a rodent in these circumstances is to explore the novel object; exploration of the novel object relative to the familiar is taken as a measure of object recognition memory. This test has many practical advantages, but its relationship to the specific cognitive domains that are compromised in schizophrenia is unclear.

#### Social Deficits

People with schizophrenia show deficits in social cognition, as well as negative schizotypy. Schizophrenia is associated with deficits in social perception, theory of mind (understanding others' mental states), emotion perception (understanding social cues), emotion processing, and social knowledge (25). General cognitive deficits accounted for a substantial portion of the variance in social perception and emotion perception, but social cognition *per se*, independently of cognitive deficits, contributed significantly to overall social function (25, 26). Social deficits were mildly correlated with negative symptoms and a lesser extent with positive symptoms (27). Social anhedonia, a core aspect of negative schizotypy, refers to reduced motivation and reward associated with social interactions. The relationship between negative schizotypy and social cognition is unclear; nevertheless, social anhedonia and impaired social functioning in general are clear risk factors for schizophrenia (28).

Alterations in social cognition domains are challenging to measure in rodents. Two basic behavioral paradigms have been employed in this regard: the Social Interaction (SI) and social recognition (SR) tests (29). In the SI test, 2 individuals that had received the same experimental treatment are placed inside an open field arena, and the number of social interactions is the principal outcome measure. The SR test is analogous to the NOR test described above, except the test stimuli are conspecifics, rather than objects. While reduced social interaction in the SI test could be interpreted as social anhedonia, it is perhaps more difficult to relate performance deficits in the SR test to specific any specific social cognition domain.

### Controlled Experimental Manipulations (Independent Variable)

Ideally, the experimental manipulation applied for inducing neurobehavioral pathology in an animal model should correspond to a known disease risk or pathogenic factor(s), and should be relatable to an existing theoretical framework for pathogenesis. The present review will focus on studies of CBD or endocannabinoid system modulation in three schizophrenia models: acute and subchronic challenge with an NMDA receptor antagonist, and neurodevelopmental models. Results of these studies are summarized in Table 1.

**Table 1:** Summary of studies on the effects of CBD or endocannabinoid system modulators on deficits in PPI, cognition, or social interaction in animal models of schizophrenia.

**Outcome measure**	**CBD**	**CB1/CB2**	**Anandamide/2-AG**	**TRPV1**	**5-HT1A**
PPI	1. **Prevention;** *Acute NMDA, mice;*(30) 2. **No effect;** *SC/C NMDA, mice;*(31) 3. **No effect;** *Acute NMDA, rat;*(32)	1. **Prevention;** Rimonabant, AM251; *Acute NMDA, rat;*(33) 2. **No effect;** Rimonabant, WIN55, 212-2; *Acute NMDA, mouse;* (34)	No studies	1. **Prevented therapeutic effect of CBD;** Capsazapine; *Acute NMDA, mice;*(30)	No studies
Cognitive	1. **Prevention;** *Acute NMDA, rat;*(35) 2. **Prevention;** *SC/C NMDA, rat;*(36) 3. **Prevention;** *SC/C NMDA, mice;*(37) 4. **Reversal;** *SC/C NMDA, mice;*(38) 5. **Reversal;** *MIA, rat* (39, 40) 6. **Reversal;** *MAM, rat* (41) 7. **No effect;** *Acute NMDA, rat* (42) 8. **Negative effect;** *Unmanipulated rat;*(35)	1. **Prevention;** AM251; *SC/C NMDA, rat;*(43) 2. **Prevention;** AM251; *Acute NMDA, mice* (44) 3. **Reversal;** AM251; *SC/C NMDA, rat;*(45) 4. **No effect;** Oleamide; *Acute NMDA, mice;*(44) 5. **No effect;** AM251 *MAM, rat* (41) 6. **No effect on therapeutic effect of CBD;** AM251, AM630; *SC/C NMDA, mice;*(38)	1. **Prevention;** URB597; *Acute NMDA, mice;*(46) 2. **No effect;** URB597; *SC/C NMDA, rat;*(45) 3.**Negative effect;** JZL184; *Acute NMDA, mice;*(46)	No Studies	1. **Prevented negative effect of CBD;** WAY100635 *Untreated rat* (35) 2. **Prevented therapeutic effect of CBD;** WAY100635 *SC/C NMDA mice;*(38)
Social	1. **Prevention (intermediate dose);** *Acute NMDA, rat;*(32) 2. **Prevention;** SC/C NMDA, mice; (37) 3. **Reversal;** *SC/C NMDA, mice;*(38) 4.**Reversal;** *MIA, rat;* (39, 40) 5. **Reversal;** *MAM, rat;*(41) 6. **No effect (high dose);** *Acute NMDA, rat;*(32) 7. **No effect;** *Acute NMDA, rat* (47) 8. **Negative effect (low dose);** *Acute NMDA, rat;*(32) 9. **Negative effect;** *Untreated rat;*(47) 10. **Prevented therapeutic effect of antipsychotic;** *Acute NMDA, rat;*(47)	1. **Reversal;** AM251; *SC/C NMDA* (48) 2. **Reversal;** AM251; *MAM, rat;*(41) 3. **No effect;** AM251; *SC/C NMDA;*(45) 4. **Negative effect;** CP55,940; *Unmanipulated rat* (48). 5. **Prevented therapeutic effect of URB597;** AM251; *SC/C NMDA, rat;*(49)	1. **Reversal;** URB597; *SC/C NMDA, rat;* (45, 49) 2. **Negative effect;** URB597; *Unmanipulated rat;* (49, 50)	1. **Prevented negative effect of URB597;** Capsazapine; *Unmanipulated rat;*(49)	1. **Prevented therapeutic effect of CBD;** WAY100635 *SC/C NMDA; mice;*(38)

*Bold typeface summarizes effect of experimental pharmacological treatment on the outcome measure. “Prevention” refers to preventing the induction of deficits by the experimental manipulation; “Reversal” refers to reversing deficits previously induced by the experimental manipulation; “Negative effect” refers to inducing or worsening deficits. In column 2, the experimental pharmacological treatment is CBD; in columns 3–6, the specific pharmacological treatment is specified for each entry. The experimental manipulation and animal species are in italic typeface: Acute NMDA antagonism (Acute NMDA), subchronic/chronic NMDA antagonism (SC/C NMDA), maternal immune activation (MIA), methylaxymethanol acetate treatment (MAM), or no experimental manipulation*.

## Experimental Modulation of the Endocannabinoid System in NMDA Receptor Antagonist and Neurodevelopmental Models of Schizophrenia

Based on reported similarities between the psychotropic effects of PCP and ketamine (glutamate NMDA receptor antagonists) and the positive, negative, and cognitive symptoms of schizophrenia, it was proposed that schizophrenia may be associated with a generalized hypofunction of NMDA receptors (51). This generalized dysfunction is believed to alter cortical information processing by altering the firing of cortical GABAergic interneurons, as well as disrupt the balance of cortical—subcortical dopamine (52). Considering this theoretical framework for schizophrenia pathophysiology, two general approaches have been taken for modeling schizophrenia in rodents: acute or subchronic/chronic challenge with NMDA receptor antagonist.

### Acute Challenge With NMDA Receptor Antagonist

Three different non-competitive NMDA receptor antagonists have been used in rodent models of schizophrenia: ketamine, phencyclidine (PCP), and MK-801. Immediate effects of NMDA receptor antagonism in human subjects are similar to acute psychosis, and experimental results derived from this model are best considered within that context. Following is a summary of results of studies of endocannabinoid system manipulation in rodent models of schizophrenia based on acute challenge with NMDA receptor antagonist, considering the outcome measures of PPI, cognitive tests, and social interaction tests.

#### PPI

In a study of male Swiss mice, 0.3 mg/kg MK-801 significantly increased the startle response and reduced PPI, and this effect was prevented by CBD (5 mg/kg). The therapeutic effect of CBD was blocked by capsazepine (a TRPV1 receptor antagonist) (30). In another recent study of male Swiss mice, PPI alterations induced by MK-801 were not prevented by rimonabant or by WIN 55,212-2 [full agonist at the cannabinoid receptor type 2 (CB2)] (34). Contrary to results obtained in male Swiss mice, in male Sprague Dawley rats CBD (3–30 mg/kg) did not prevent MK-801-induced PPI deficits (32), and rimonabant or AM251 (CB1 antagonist) prevented PPI deficits induced by MK-801 and PCP (33).

#### Cognitive Deficits

In male Swiss mice, MK-801 induced deficits in memory acquisition, consolidation, and retrieval in the passive avoidance task that were prevented by AM 251, but not by oleamide (CB1 agonist) (44). Inhibitors of enzymes that degrade anandamide or 2-arachidonoylglycerol (2-AG), respectively, reduced and augmented cognitive effects of acute MK-801 challenge in this task (46). In male hooded Lister rats, MK-801 administered 30 min prior to testing induced working memory deficits in a delayed matching to position task; a crude CBD extract administered concomitantly with MK-801 failed to significantly reduce this effect (42). In male Sprague Dawley rats, MK-801 injected directly into the prefrontal cortex (PFC) induced deficits in attentional set-shifting, which were prevented by co-injection of CBD. CBD administered alone had an effect similar to MK-801, and this effect was prevented by concurrent infusion of a 5-HT1A/7 antagonist (35). Notably, the cognitive tasks used in these studies can be related to specific cognitive domains that are altered in schizophrenia (working memory, declarative memory and attentional set shifting).

#### Social Deficits

Acute MK-801 challenge (0.6 mg/kg) to male Sprague Dawley rats reduced the number of social encounters in the SI test (32), and a low dose of either CBD (3 mg/kg) or clozapine (1 mg/kg) prevented this effect, while higher doses (30 and 10 mg/kg, respectively) had no effect, nor did CBD given alone. In a separate study, CBD (3 mg/kg) prevented the effects of MK-801 (0.3 mg/kg) on social encounters, while a lower dose of CBD (1 mg/kg) potentiated the effects of MK-801 (53). In male Wistar rats, MK-801 (0.03–0.15 mg/kg) had no effect on social motivation, but social memory was impaired. This impairment was reduced by aripiprazole (an antipsychotic; 2 mg/kg), while risperodone and olanzapine had no effect. CBD did not prevent MK-801-induced deficits in social memory, and at high doses (12 and 30 mg/kg) impaired it. When CBD was given with aripiprazole, the effect of aripiprazole was lost (47). Methodological differences between these two groups—both with respect to experimental manipulation and outcome measures—make comparisons somewhat difficult, but it appears that MK-801 might have distinct dose-dependent effects on social behavior and social memory, and CBD, in turn, has distinct effects according to dose, outcome measure, and pharmacological context.

### Subchronic/Chronic Challenge With NMDA Receptor Antagonist

Subchronic or chronic administration of ketamine, PCP, or MK801 has neurobehavioral effects that persist after drug washout. Many of these effects are similar to pathological characteristics of schizophrenia, including a reduction in hippocampal parvalbumin positive GABAergic interneurons, which is also observed in the prefrontal cortex in schizophrenia (54–58). Thus, repeated administration of NMDA receptor antagonists may replicate in rodents important neurobiological alterations of schizophrenia.

#### PPI

There is one published study on the effects of CBD on PPI deficits induced by chronic MK-801 treatment. MK-801 (0.1, 0.5, or 1.0 mg/kg) was administered across 14, 21, or 28 days, to 6 week old male C57/BL/6J mice. CBD (15, 30, 60 mg/kg) or clozapine (1 mg/kg) was co-applied beginning on day 6 of treatment. MK801 (1 mg/kg) for 28 days impaired PPI (measured 1 day after final MK-801 injection) and coadministration of either 60 mg/kg CBD or 1 mg/kg clozapine prevented this effect (31).

#### Cognitive Deficits

When MK-801 (0.5 mg/kg) was administered twice per day across 14 days to adult male C57BL/6J mice, deficits were observed in the NOR test 1 week after the final dose of MK801. These deficits were reversed by clozapine (1 mg/kg/day) or CBD (15 and 30 mg/kg/day, but no effect of 60 mg/kg/day), which were administered for 7 days following MK-801 treatment. The effects of CBD were prevented by the co-administration of WAY100635 (5-HT1A/7 antagonist), but not by co-administration of AM251 or AM630 (CB2 antagonist) (38). Gomes et al. (37), using the same chronic MK-801 treatment protocol applied to male C57/BL/6J mice described in the previous paragraph, reported that chronic MK-801-induced deficits in the NOR test were prevented by either co-administration of clozapine or 60 mg/kg CBD. In a separate study, adolescent male Lister-Hooded rats were treated for 5 successive days with PCP, followed by intermittent administrations across the next 3 weeks. Deficits in novel object discrimination were observed 3 days after the final PCP administration; these deficits were prevented by clozapine or AM251 (5 and 0.5 mg/kg, respectively, co-administered with PCP beginning on treatment day 10). PCP treatment increased 2-AG, but not anandamide levels in the prefrontal cortex, while AM251 increased anandamide levels when administered alone or when co-administered with PCP (43). Similarly, chronic ketamine administration (30 mg/kg/day for 10 days) to adult male Sprague Dawley rats caused deficits in the NOR test observed 7 or 14 days after the final dose of ketamine, while acute CBD administration (7.5 mg/kg just prior to the first NOR test) or subchronic CBD administration (7.5 mg/kg/day for 7 successive days) reversed this effect (36).

A single study (45) using this model and applying a cognitive test paradigm recommended by CNTRICS reported that PCP treatment (5 mg/kg, 2 times per day, for 7 days) in adult male Wistar rats caused a working memory deficit in a delayed alternation task 5 days after the final PCP dose. This deficit was reversed by AM251 (1 mg/kg), but not URB597 (an inhibitor of FAAH; 0.3 mg/kg), administered just before the task. Interestingly, both AM251 and URB597 caused working memory deficits when administered to rats that had not received PCP treatment. Taken together, studies of cognition in these models showed that deficits in object recognition and working memory were reversed and/or prevented by clozapine, AM251, or CBD. Therapeutic effects of CBD may be mediated by the 5-HT1A receptor. Notably, anandamide levels, or the relationship between anandamide and 2-AG levels, appeared to be associated with positive treatment response.

#### Social Deficits

In the studies of Rodrigues da Silva et al. (38) and Gomes et al. (37) described above, chronic MK-801 treatment resulted in reduced social interaction in male C57BL/6J mice that, along with cognitive deficits, were mitigated by either clozapine or CBD. The effect of CBD was prevented by WAY100635 administration (38). Likewise, in the Seillier et al. (45) study described above, subchronic PCP treatment reduced social interaction between two freely interacting individuals that had received the same experimental treatment, in addition to inducing object memory deficits. However, unlike the cognitive deficits, social deficits were reversed by URB597, but not by AM251. In a later study by Seillier et al. (49) (same protocol as in 2010), it was reported that URB597 again reversed the social deficits induced by PCP, but induced social deficits in saline treated rats, a result that was replicated in a separate study (50). In PCP treated rats, the positive effect of URB597 was prevented by AM251, while the negative effect of URB597 in saline treated rats was prevented by capsazepine (a TRPV1 receptor antagonist). These results suggest that the positive effects of increasing anandamide levels within a pathological system may be mediated by the CB1 receptor, while negative effects of this same manipulation within a healthy system may be mediated by the by TRPV1 receptor. These investigators hypothesize that deficient CB1 receptor stimulation as a consequence of chronic PCP treatment is responsible for social withdrawal. Seillier and Guiffrida (48) next applied a modified versión of the social interaction test in which the focal animal interacted with untreated stimulus animals confined to wire mesh cages within the testing arena. This protocol allowed for distinguishing between social motivation and social recognition memory. PCP treated rats did not show differences from saline with respect to social motivation, but did show deficits in the capacity to distinguish between familiar and unfamiliar conspecifics. This effect of PCP was reversed by AM251, while in saline-treated rats, a CB1 agonist (CP55,940) induced social recognition deficits.

### Neurodevelopmental Models

Maternal infection during pregnancy by certain viral pathogens is known to be a significant risk factor for schizophrenia in the offspring. Thus, risk for schizophrenia is increased by 2–7-fold from influenza infection during the first half of pregnancy (59). Several animal models have been developed in order to reproduce this risk factor; in general, these models involve gestational exposure to specific antigens that induce an immune response mimicking an infection (60). One such model that has been applied in the present context involves exposing the pregnant female rodent to polyinosinic:polycytidylic acid (Poly I:C), which is a compound structurally similar to viral RNA that stimulates an immune response similar to a viral infection. Notably, the results of these studies coincide well with those obtained using models based on NMDA receptor antagonism.

Osborne et al. (39, 40) applied a single dose of Poly I:C to pregnant Sprague Dawley rats on gestational day 15. Beginning on postnatal day 56, the adult male (39) and female (40) offspring were treated daily with CBD (10 mg/kg) or vehicle, and across days 72–80 were subjected to a NOR test, a delayed alternation test for working memory, and a social interaction test. Maternal immune activation resulted in deficits in object recognition and working memory, and decreased social interaction. All of these effects of maternal immune activation were reversed by CBD treatment.

A second neurodevelopmental model involves treating the pregnant dam with methylaxymethanol acetate (MAM), a DNA methylating agent, on gestational day 17. This treatment results in a number of neurobiological alterations in the adult offspring similar to those seen in schizophrenia (61). Stark et al. (41) found that CBD (30 mg/kg, but not 10 mg/kg) administered across postnatal days 19–39 reversed MAM-induced deficits in novel object discrimination in the NOR test as well as social interaction deficits. Interestingly, while neither AM251 nor haloperidol administered across the same postnatal period reversed NOR deficits, AM251 reversed social interaction deficits, and this effect was accompanied by a decrease in 2-AG levels in the prefrontal cortex.

## From the Clinic to the Lab and Back Again

Clinical trials suggest that CBD may be a useful pharmacotherapy for schizophrenia, but more studies are needed. Some clinical variables that are important to consider in future studies are disease chronicity (first episode vs. chronic disease), details of drug administration (alone or in combination with antipsychotics), and therapeutic objective [reducing existing symptoms or preventing disease progression; (62, 63)]. Likewise, in pre-clinical studies, these variables should be systematically considered within the experimental design. For example, acute NMDA receptor antagonist treatment can model the acute psychotic episode, while recent onset or chronic disease are best represented by persistent effects of neurodevelopmental challenges or subchronic or chronic administration of NMDA antagonists. CBD should be tested alone and in combination with distinct antipsychotics. [Indeed, at least one study indicates that CBD may reduce the effectiveness of antipsychotic treatment in an animal model (47)]. Possible preventative effects of CBD can be investigated by administering CBD before or along with the experimental challenge. More attention must be given to the comparability of the independent variables defined in animal model studies to controllable (and uncontrollable) clinical variables such as those mentioned above.

Likewise, outcome measures (dependent variables) should be standardized between pre-clinical and clinical studies. The present mini review discussed some outcome measures that are reasonably comparable between human and animal studies, and that have been applied in the laboratory (PPI and specific cognitive tests). The NOR, SI, and SR tests are highly practical for large scale use, but homologous tests for the clinic have not been developed. With respect to cognitive deficits, rodent tests that have clear human homologs, such as those identified by the CNTRICS initiative, should be more frequently applied. With respect to social deficits, new behavioral tests should be designed that more clearly capture specific domains of social dysfunction, perhaps including objective observations of human subjects in controlled social situations.

The body of studies presented here illustrates the general lack of comparability between pre-clinical and clinical studies. Of the clinical studies, only one (8) is reasonably comparable to just 4 (of 19) pre-clinical studies with respect to independent variables: CBD administered as a single drug to subjects with existing acute schizophrenia is comparable to CBD administration to rodent models that received subchronic/chronic NMDA receptor antagonist followed by drug washout (36, 38) or to neurodevelopmental models (39–41). Nevertheless, the outcome measures in these pre-clinical studies are not easily comparable to those of the Leweke et al. (8) study, except for deficits in social interaction, which are contemplated in the PANSS negative symptom subscale.

What are some other factors that may limit the translatability of pre-clinical results to the clinic? In the specific case of CBD and endocannabinoid modulators, responsiveness may be highly sensitive to dose. In the relatively few animal model studies where several different doses of CBD were tested, there was some indication that dose responsiveness to CBD might take the form of an inverted U, where lower and higher doses are ineffective or have negative effects. Given this information, it seems important that body mass be taken into consideration when analyzing clinical results, perhaps by including this factor as a covariable in the statistical analysis of the results. Secondly, clinical populations are likely heterogeneous with respect to underlying pathophysiologies, perhaps some being responsive to CBD or endocannabinoid system modulators, and others unresponsive. With this in mind, it might be useful to examine in an exploratory manner risk factor profiles of patient responders vs. non-responders, in order to define the dependent variables that are most appropriate for modeling responsiveness (and non-responsiveness) to CBD or endocannabinoid system modulators.

It is striking that the vast majority pre-clinical studies reviewed here were carried out using exclusively male animals. Human clinical populations obviously comprise both sexes, not to mention being more diverse than laboratory animals in terms of underlying pathology, age, diet, body weight, among many other characteristics. By contrast, laboratory rodent strains have been selectively bred for hundreds of generations and raised under highly controlled conditions, no doubt reducing genetic and epigenetic variability of the population. All of these factors could introduce variability into the clinical response that is not represented in pre-clinical studies, thus limiting the predictive power of the latter.

Since the results of clinical studies on a homogenous population would be almost meaningless in clinical practice, one possible way to address this limitation may be to systematically carry out pre-clinical experiments in mixed-sex animal populations that have more inherent genetic diversity, perhaps including rodent or non-rodent species that have not had an extended history of laboratory rearing, such as the prairie vole or deer mouse. Since important interspecies differences may exist with respect to pharmacological responsiveness [for example, rodent FAAH is inhibited by CBD, while the human counterpart is not (9)], it is advisable to investigate such details in a number of different species. In addition, a broader range of models should be employed: models based on different risk factors (distinct experimental manipulations) should be simultaneously tested, perhaps within the same experiment or through a coordinated multicenter effort, with the goal of approaching the diversity of the clinical population and potentially improving the predictive power of pre-clinical studies. Clearly, this strategy would require larger cohort sizes and strict multicenter coordination with regards to experimental design and outcome measures, the latter of which should be uniformly applied and readily comparable to outcome measures in clinical studies.

Animal models present an opportunity to examine in detail factors that might influence the therapeutic response to CBD in the clinic, as well as the physiological and neurobiological underpinnings of this response. In the case of cannabinoids or pharmacological manipulation of the endocannabinoid system as possible means to treat schizophrenia, studies on animal models indicate that CBD may be a useful pharmacotherapy, and suggest that pharmacotherapies that modulate anandamide and 2-AG levels should be developed and explored. Finally, studies on animal models point to interactions between cannabinoid receptors, the 5-HT1A/7 receptor, and the TRPV1 receptor that should be explored as possible targets for pharmacotherapy.

## Author Contributions

KH: conceptualized, researched, and wrote the present article.

## Conflict of Interest

The author declares that the research was conducted in the absence of any commercial or financial relationships that could be construed as a potential conflict of interest.
